# Comparisons of tumor-infiltrating lymphocyte levels and the 21-gene recurrence score in ER-positive/HER2-negative breast cancer

**DOI:** 10.1186/s12885-018-4228-6

**Published:** 2018-03-24

**Authors:** Sung Gwe Ahn, Yoon Jin Cha, Soon June Bae, Chanik Yoon, Hak Woo Lee, Joon Jeong

**Affiliations:** 1 Department of Surgery, Gangnam Severance Hospital, Yonsei University College of Medicine, Seoul, Korea; 2 Department of Pathology, Gangnam Severance Hospital, Yonsei University College of Medicine, Seoul, Korea

**Keywords:** Tumor-infiltrating lymphocytes, 21-gene recurrence score, Breast cancer

## Abstract

**Background:**

Recent studies have shown that tumors with extensive tumor-infiltrating lymphocytes (TILs) have a higher probability of pathologic complete response, even in luminal/human epidermal growth factor 2 (HER2)-negative breast cancer. We compared TIL levels and the 21-gene recurrence score (RS) in estrogen receptor (ER)-positive/HER2-negative breast cancer.

**Methods:**

We evaluated the percentage of stromal TILs in 198 ER-positive/HER2-negative patients in whom RS was obtained by examining slides of surgical specimens by standardized methodology proposed by the international TIL Working Group. TIL levels were categorized as high (≥ 60%), intermediate (11–59%), or low (≤ 10%). All tumors were treatment-naïve.

**Results:**

Ninety-seven (49.0%), 88 (44.4%), and 13 patients (6.6%) had low, intermediate, and high TIL levels, respectively. There was a significant but weak correlation between continuous RS and continuous TIL levels (Pearson’s *R* = 0.201, *p* = 0.004). The mean RS was significantly highest in high TIL tumors (17.8 ± 10.7 in low TIL tumors, 19.4 ± 8.7 in intermediate TIL tumors, and 26.2 ± 8.2 in high TIL tumors; *p* = 0.014). However, when we compared categorized RS and TIL levels, we found that tumors with high TIL levels tended to have higher RS (≥ 26) but it was not significant (*p* = 0.155). Furthermore, multivariate analysis revealed that high RS was not an independent factor associated with high TIL levels. Chemo-endocrine therapy was more frequently performed among patients with high TILs and less frequently among those with low or intermediate TILs (*p* <  0.001).

**Conclusions:**

Despite of a weak correlation between continuous TIL levels and RS, we found that tumors with high TIL levels tended to have a higher RS in ER-positive/HER2-negative breast cancer. Further study is warranted considering the clinical outcomes.

## Background

The presence of tumor-infiltrating lymphocytes (TILs) reflects an adaptive anti-tumor immune response [1]. Although the immunogenicity of breast cancer has not been strongly considered in research or clinical practice, TILs are emerging as biomarkers for predicting clinical response to chemotherapy for breast cancer [2–8]. Recent translational studies from large prospective trials suggest that tumors with high TIL levels show a superior prognosis compared to tumors with low TIL levels in breast cancer patients treated with neoadjuvant or adjuvant chemotherapy [2–8]. The correlation between high TIL levels and improved clinical outcome was most prominent in triple-negative breast cancer (TNBC) [2, 6–8]. After the initial report from the BIG2–98 trial, a positive relationship between TIL levels and clinical outcome in TNBC was validated in independent cohorts in two clinical trials [2, 6].

Interestingly, TIL levels have not been shown to be prognostic in patients with estrogen receptor (ER)–positive tumors receiving adjuvant chemotherapy. However, a recent study reported that patients with a high level of TILs have a greater chance of obtaining a pathological complete response (pCR), even in hormone receptor (HR)-positive/human epidermal growth factor receptor 2 (HER2)-negative breast cancers [9]. In addition, a recent meta-analysis showed that HR-positive/HER2-negative tumors with high TIL levels at baseline biopsy, prior to chemotherapy, have a higher probability of pCR (*n* = 1366) [10].

The 21-gene recurrence score (RS) is derived from a polymerase chain reaction-based test that examines 16 tumor related genes and 5 reference genes to analyze cell proliferation, estrogen signaling, and invasion capacity. It has been shown to predict the clinical benefit of chemotherapy for individuals with ER-positive/HER2-negative breast cancer [11, 12]. Furthermore, previous studies have shown that tumors with a high RS have a higher rate of pCR in the neoadjuvant setting [13, 14].

Both TIL levels and the RS could serve as biomarkers associated with chemotherapy responsiveness in HR-positive/HER2-negative breast cancer. Moreover, identifying suitable patients among those with HR-positive/HER2-negative cancers who derive clinical benefit from chemotherapy has been a critical issue; however, the relationship between the two markers has not been intensively examined.

In this study, we examined the association between TIL levels and RS in patients with ER-positive/HER2-negative breast cancer who did not receive neoadjuvant chemotherapy (NAC).

## Methods

### Patients

Between August 2011 and April 2017, eligible patients were identified from the breast cancer database of the Gangnam Severance Hospital [15]. During the period, 212 patients underwent Oncotype DX testing. All patients had ER-positive/HER2-negative breast cancer and did not undergo NAC. In 198 patients, TIL levels were successfully evaluated using treatment-naïve surgical specimens. A pathologist (YJC) reviewed the histologic features by using Hematoxylin & eosin (H&E)-stained slides for all cases. Histologic grade for breast cancer was assessed using the Nottingham grading system [16]. The institutional review board of the Gangnam Severance Hospital, approved the study to be in accordance with guidelines of good clinical practice and the Declaration of Helsinki. The need for informed consent was waived under the approval of the institutional review board due to the retrospective design.

### Assessment of TIL levels

Stromal TIL levels were scored according to the guidelines for TIL assessment by the TILs Working Group [1]. H&E-stained whole tumor slides were examined and scored by a pathologist (YJC). Briefly, the tumor area as defined by the presence of invasive tumor was evaluated, and all mononuclear cells, including lymphocytes and plasma cells but not polymorphonuclear leukocytes, were scored. Areas outside of the tumor border, around the intraductal component, and normal lobules were excluded. Within the tumor border, TILs with crush artifacts and necrosis were excluded. For each case, three representative tumor areas were evaluated for TILs, and the average score was reported as a percentage. TIL levels was categorized as high (≥ 60%), intermediate (11–59%), or low (≥ 10%). The cut-off point was defined using the criteria suggested by Denkert et al. [10].

### Immunohistochemistry

For immunohistochemistry (IHC), antibodies specific for ER (1:100 clone 6F11; Novocastra, Newcastle upon Tyne, UK), progesterone receptor (PR; clone 16; Novocastra), HER2 (4B5 rabbit monoclonal antibody; Ventana Medical Systems, Tucson, AZ, USA), and Ki-67 (MIB-1; Dako, Glostrup, Denmark) were stained using formalin-fixed, paraffin-embedded tissue sections [17]. Positivity of ER and PR IHC expression was defined according to the modified Allred system: positive, Allred score 2–8; and negative, Allred score 0–1 [18]. All tumors included in this study had ER positivity (Allred scores ≥3). HER2 status was considered positive with a score of 3+ and negative with a score of 0 or 1+ [19]. Tumors with a score of 2+ underwent fluorescent in situ hybridization analysis according to the manufacturer’s instructions (PathVysion kit; Vysis, Downers Grove, IL, USA or HER2 inform; Ventana) [19]. Ki-67 expression was evaluated by YJC and displayed presented as a percentage (range 0–100%) of positive tumor cells.

### Oncotype dx assays

The RS is calculated by the Oncotype Dx assay [11, 12]. RS scores are calculated on a scale from 0 to 100 and derived from the reference-normalized expression measurements of 16 cancer-related genes (Ki67, STK15, Survivin or BIRC5, CCNB1 or cyclin B1, MYBL2, GRB7, HER2, ER, PGR, BCL2, SCUBE2, MMP11 or stromelysin 3, CTSL2 or cathepsin L2, GSTM1, CD68, and BAG1) and five reference genes. Quantitative single gene scores are determined via reverse transcriptase-polymerase chain reaction. Expression of each gene was measured in triplicate and normalized to a set of five reference genes (beta-actin [ACTB], GAPDH, GUS, RPLPO, and TFRC). Reference-normalized expression measurements ranged from 0 to 15, where a one-unit increase approximately reflects a two-fold increase in RNA.

Tumors were classified into the following categories: low risk (RS < 18), intermediate risk (RS 18–30), and high risk (RS ≥ 31). The RS was also dichotomized with a cutoff of 26 as in the TAILORX trial [20]. The Oncotype Dx assay was supplied by Genomic Health (Redwood City, CA, USA) and performed using RNA extracted from formalin-fixed paraffin-embedded tissue. After a review of hematoxylin and eosin-stained slides to determine whether sufficient invasive breast cancer was present and whether manual microdissection was indicated, RNA was extracted from the unstained sections. Cases without tumor samples (i.e., depleted by prior tissue studies) or with cancer cells occupying < 5% of the section area were excluded from the assay [11]. All tissues from patients in this study were successfully analyzed.

### Statistical analysis

The primary objective of this study was to test the correlation between RS and TIL levels. Pearson’s R was calculated to measure the correlative value between two continuous scores. Discrete variables were compared using the χ2 test. Continuous variables were compared using the Mann-Whitney U and Kruskal-Wallis tests. Student’s t-test or one-way analysis of variation (ANOVA) was used to compare means. The variables with statistical significance in the univariate analysis were included in the selection of the full multivariate model, and backward elimination was taken to arrive at the final model.

The Kolmogorov-Smirnov test was applied to test the normal distribution of continuous variables, including RS and TIL levels. The distributions of nonparametric variables were compared using the Kruskal-Wallis test. A post-hoc test was performed using the Bonferroni-corrected Dunn’s procedure. SPSS version 18 (SPSS Inc., Chicago, IL, USA) was used to perform the statistical analyses. Statistical significance was defined as a *p*-value < 0.05.

## Results

### Baseline characteristics

One hundred ninety-eight patients with ER-positive/HER2-negative tumors were included in the analyses. Ninety-eight (50%), 80 (40%), and 20 patients (10%) had a low, intermediate, and high RS, respectively, while 97 (49%), 88 (44%), and 13 patients (7%) had low, intermediate, and high TIL levels, respectively. The baseline characteristics are presented in Table 1. The median age of these patients was 49 years (range: 27–75 years). Thirty-four patients had node-positive disease, and 3 had micrometastasis. No patient in the study population had a stage higher than IIB.

**Table 1:** Baseline characteristics

	Low TIL (*N* = 97)	Intermediate TIL (*N* = 88)	High TIL (*N* = 13)	P-value ^a^
Age, median (range)	50 (28–75)	48 (34–74)	50 (27–73)	0.280 ^**b**^
Histology				0.152
IDC	73 (75.3)	75 (85.2)	13 (100.0)	
ILC	14 (14.4)	6 (6.8)	0 (0.0)	
Others	10 (10.3)	7 (8.0)	0 (0.0)	
pT stage				0.540
T1	65 (67.0)	61 (69.3)	7 (53.8)	
T2	32 (33.0)	27 (30.7)	6 (46.2)	
pN stage				0.286
N0	84 (86.6)	70 (79.5)	10 (76.9)	
N1mi	5 (5.2)	3 (3.4)	0 (0.0)	
N1	8 (8.2)	15 (17.0)	3 (23.1)	
pStage				0.210
IA	55 (56.7)	50 (56.8)	6 (46.2)	
IB	4 (4.2)	2 (2.3)	0 (0.0)	
IIA	37 (38.1)	30 (34.1)	5 (38.5)	
IIB	1 (1.0)	6 (6.8)	2 (15.4)	
Nuclear grade ^c^				< 0.001
1	4 (4.1)	5 (5.7)	1 (7.7)	
2	79 (81.5)	56 (63.6)	1 (7.7)	
3	12 (12.4)	27 (30.7)	11 (84.6)	
Unknown	2 (2.0)	0 (0)	0 (0)	
Histologic grade ^**c**^				0.001
I	15 (15.5)	21 (23.9)	0 (0)	
II	71 (73.2)	57 (64.8)	7 (53.8)	
III	9 (9.3)	10 (11.4)	6 (46.2)	
Unknown	2 (2.0)	0 (0)	0 (0)	
PR ^d^				0.649
Positive	81 (83.5)	76 (86.4)	10 (76.9)	
Negative	16 (16.5)	12 (13.6)	3 (23.1)	
Ki-67 ^c^				0.506
≥ 20%	18 (18.6)	15 (17.1)	4 (30.8)	
< 20%	79 (81.4)	72 (81.8)	9 (69.2)	
Unknown	0 (0.0)	1 (1.1)	0 (0.0)	

^a^χ2 test except ^b^

^b^Kruskal-Wallis test

^c^Missing value

^d^Positive, Allred score 2–8; Negative, Allred score 0–1

Data are presented as n (%)

*IDC*: invasive ductal carcinoma, *ILC*: invasive lobular carcinoma, *pN stage*: pathologic nodal stage, *PR*: progesterone receptor, *pStage*: pathologic stage, *pT stage*: pathologic tumor stage, *TIL*: tumor infiltrating lymphocyte

### Clinical and pathological characteristics associated with TIL levels

Tumors with higher nuclear and histologic grade had high TIL levels (*p* <  0.001 and *p* = 0.001, respectively). Other characteristics, including tumor burden, PR expression, and Ki-67 expression, were not associated with TIL levels.

### Correlation between continuous RS and continuous TIL levels

**Fig. 1:**
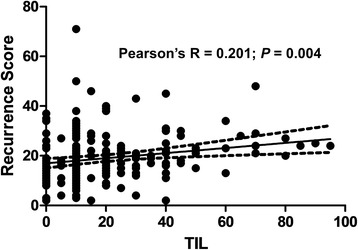
Scatter plots of continuous recurrence scores and continuous tumor infiltrating lymphocytes (TILs) (Pearson’s *R* = 0.201; *P* = 0.004)

Pearson’s R test was performed to explore the relationship between continuous RS and continuous TIL levels. A weak but significant correlation was observed between the two continuous parameters (Pearson’s *R* = 0.201; *p* = 0.004; Fig. 1).

### Comparisons categorized RS and categorized TIL levels

**Fig. 2:**
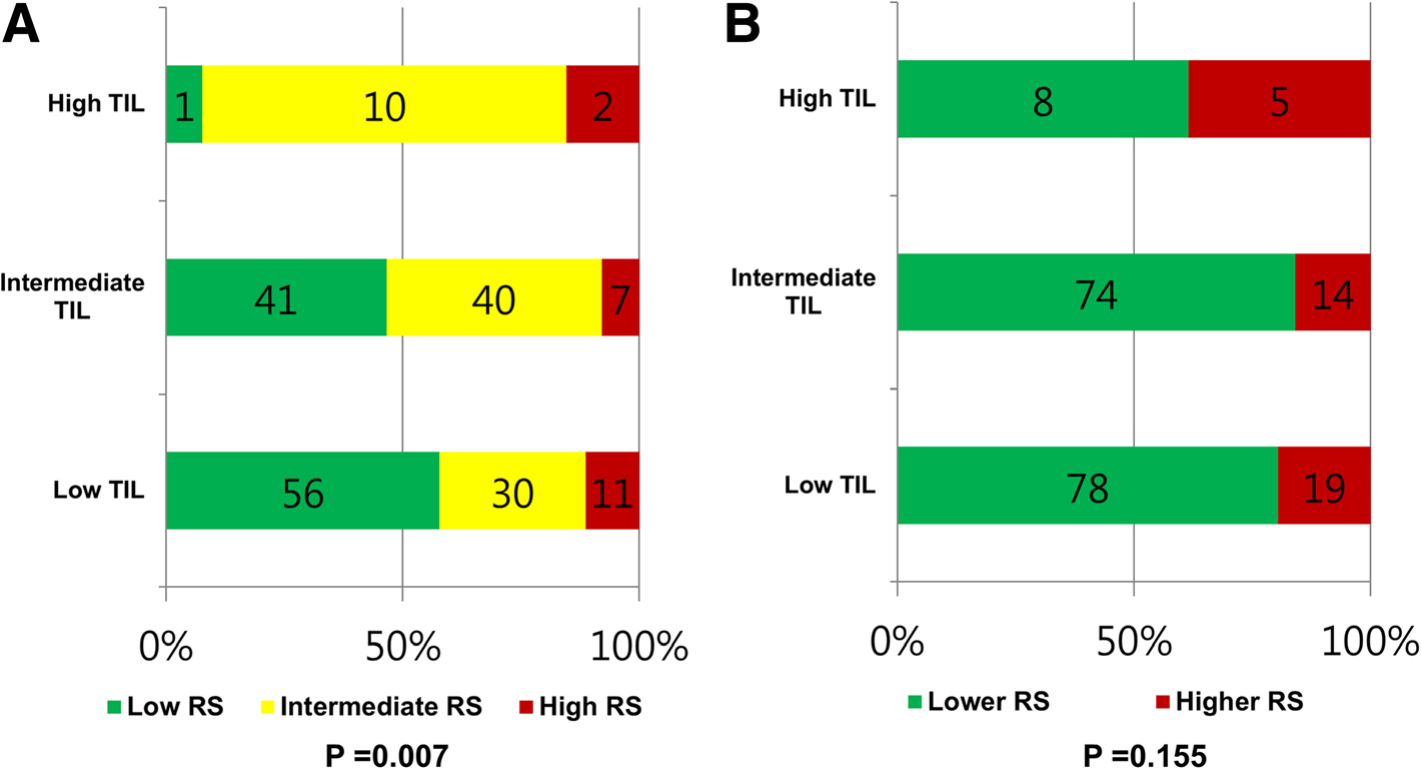
Distribution of the 21-gene recurrence score (RS) groups according to tumor infiltrating lymphocyte (TIL) count. **a** Patients with intermediate-TIL tumors showed a distribution of categorized RS similar to that of patients with low-TIL tumors. In contrast, among the patients with high-TIL tumors, the percentage of intermediate RS was significantly higher (*p* = 0.007). **b** Tumors with higher RS (≥ 26) tended to have a higher rate of high TIL levels, but it was not significant (*p* = 0.155)

Of the 97 patients with low-TIL tumors, 56 (57.7%) had a low-RS tumor, 30 (30.9%) had an intermediate-RS tumor, and 11 (11.3%) had a high-RS tumor (Fig. 2a). Eighty-eight patients with intermediate-TIL tumors showed a similar distribution of categorized RS compared to patients with low-TIL tumors (Fig. 2a). In contrast, among the patients with high-TIL tumors, the percentage of intermediate RS was 76.9%, which was significantly higher than that in patients with low- or intermediate-TIL tumors (*p* = 0.007). When we compared TIL levels with dichotomized RS, tumors with high TIL levels tended to have higher RS (≥ 26), but it was not significant (Fig. 2b; *p* = 0.155).

**Fig. 3:**
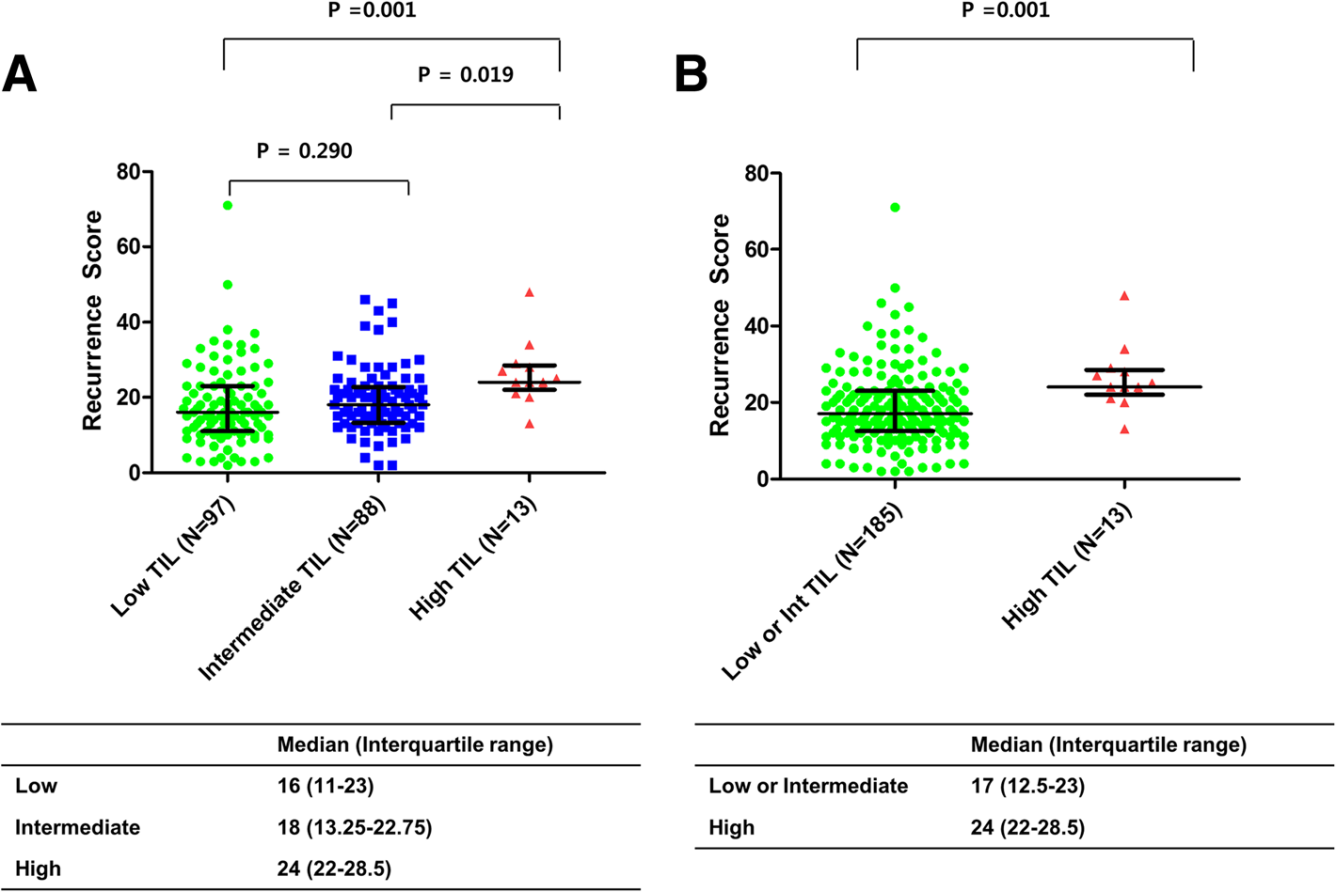
Distributions of recurrence score (RS) according to tumor infiltrating lymphocyte (TIL) levels. **a** Distributions of RS differed significantly among the three groups, which were categorized by TIL levels (*p* = 0.001 with Kruskal-Wallis test). The median RS (interquartile) was 16 (11–23) in the low-TIL group, 18 (13.25–22.75) in the intermediate-TIL group, and 24 (22–28.5) in the high-TIL group. A post-hoc test with the Bonferroni-corrected Dunn’s procedure showed that high-TIL tumors had significantly higher median RS than low- or intermediate TIL tumors (*p* = 0.001 and *p* = 0.019, respectively), whereas intermediate-TIL tumors did not have a higher median RS than low-TIL tumors (*p* = 0.290). **b** Distributions of RS differed significantly according to the dichotomized TIL levels (*p* < 0.001 with Mann-Whitney U test). The median RS was 17 (12.5–23) in the low- and intermediate-TIL group and 24 (22–28.5) in the high-TIL group. *Lines indicate the median values, and error bars indicate interquartile ranges*

Next, we compared RS distributions according to categorized TIL levels because both markers were not normally distributed (*p* < 0.001 with Kolmogorov-Smirnov test). The RS distributions differed significantly according to TIL levels (*p* < 0.001 with Kruskal-Wallis test; Fig. 3a). A post-hoc test using the Bonferroni-corrected Dunn’s procedure showed that high-TIL tumors had significantly higher median RS than low- or intermediate TIL tumors (*p* = 0.001 and *p* = 0.019, respectively; Fig. 3a). However, the RS medians did not differ significantly between low- and intermediate-TIL tumors (*p* = 0.290, Fig. 3a).

When TIL levels were divided into two groups (low- or intermediate-TILs versus high TILs), high-TIL tumors had significantly higher median RS values than low- or intermediate-TIL tumors (Fig. 3b, *p* < 0.001 with Mann-Whitney U test).

### Logistic regression analysis to identify high TIL levels

We tried to identify factors associated with high TIL levels in ER-positive/HER2-negative tumors. Variables with *p* < 0.05 on univariate analysis, including nuclear grade, histologic grade, and RS, were entered as input variables in multivariate analysis in order to distinguish factors associated with high TIL levels. Multivariate analysis revealed that nuclear grade remained the only independent variable associated with high TIL level (Table 2). Nuclear grade demonstrated the highest odds ratio (OR = 15.025; 95% confidence interval = 2.839–79.510) for predicting high TIL levels on the multivariate analysis.

**Table 2:** Binary logistic regression analysis to identify predictive factors for high TIL level

	Univariate			Multivarite		
	*P*-value	OR	95% CI	*P*-value	OR	95% CI
Nuclear grade	< 0.001			0.001		
1 or 2		Ref			Ref	
3		20.308	4.321–95.452		14.950	2.841–78.656
Histologic grade	0.001			0.379		
1 or 2		Ref			Ref	
3		7.857	2.381–25.931		1.882	0.460–7.702
Recurrence Score	0.047			0.750		
Low or intermediate		Ref			Ref	
High		2.896	1.001–9.360		1.251	0.315–4.973

*TIL*: tumor infiltrating lymphocyte, *OR*: odds ratio, *CI*: confidence interval

### Association of TIL levels with RS-guided adjuvant therapy

**Fig. 4:**
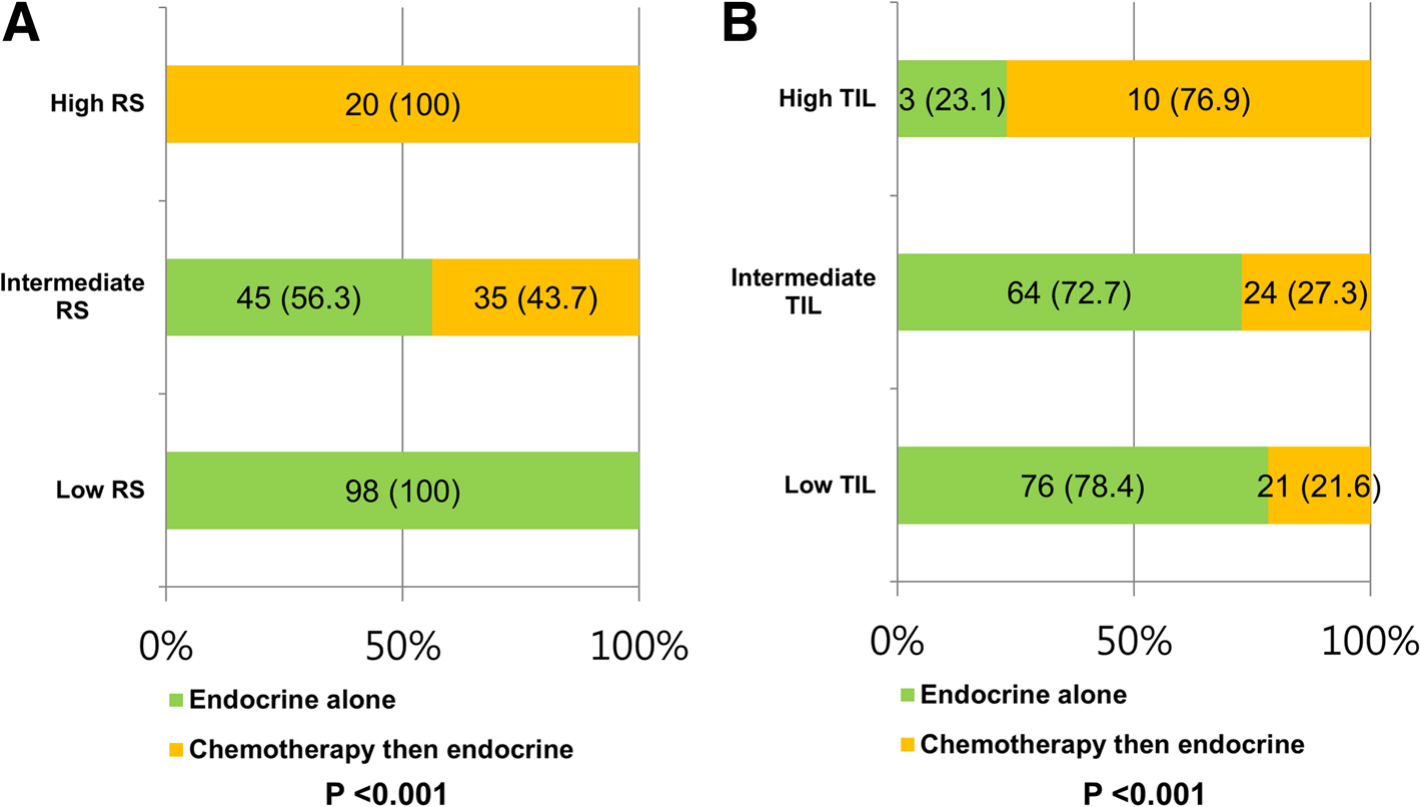
Association of TIL levels with RS-guided adjuvant therapy. **a** The proportions of adjuvant treatments according to RS (*p* < 0.001). **b** The proportions of adjuvant treatments according to TIL levels (*p* < 0.001)

Lastly, we investigated an association of TIL levels with adjuvant treatment. Based on the RS results, patients were guided to receive either endocrine therapy (ET) alone or chemo-endocrine therapy. As a result, all patients with low RS (*n* = 98) received ET alone, while all patients with high RS (*n* = 20) received chemo-endocrine therapy (Fig. 4a). Among 80 patients with intermediate RS, 45 (56.3%) were guided to forego ET alone.

Among patients receiving RS-guided treatments, associations between TIL levels and adjuvant treatments were analyzed. The classification by TIL levels was associated with a significant change in the chemo-endocrine treatment rate (*p* < 0.001, Fig. 4b). Chemo-endocrine therapy was more frequently performed among patients with high TILs (chemo-endocrine therapy in 10 [76.9%] and ET alone in 3 [23.1%]) and less frequently among those with low TILs (chemo-endocrine therapy in 24 [27.3%] and ET alone in 64 [72.7%]) or intermediate (chemo-endocrine therapy in 21 [21.6%] and ET alone in 76 [78.4%]).

## Discussion

Many investigators have extensively studied the clinical value of TILs or RS in breast cancer; however, there are only a few studies regarding an association between Oncotype DX-RS and TILs in a specific subset of breast cancer, ER-positive/HER2-negative breast cancer.

We previously hypothesized that tumors with extensive lymphocytic infiltration might have high RS because patients with high TIL levels have a higher rate of pCR to preoperative chemotherapy. Additionally, ER-positive/HER2-negative patients with high RS derive clinical benefit from adjuvant chemotherapy. If a positive correlation could be found between the two markers, further studies testing TIL levels as a biomarker that predicts clinical benefit for adjuvant chemotherapy in ER-positive/HER2-negative cancer would be warranted.

In this study, we found a weak correlation between RS and TIL levels, wherein tumors with high TIL levels tended to have a higher RS. Moreover, high-TIL tumors had significantly higher median RS than low- or intermediate TIL tumors. A weak correlation between the two continuous markers was also observed. However, a close association between categorical RS and TIL levels was not observed. In addition, multivariate analysis revealed that RS was not an independent factor associated with high TIL levels.

These biomarkers are both associated with aggressive tumor biology, but they depend on different pathways in tumor progression. RS mainly reflects the biological characteristics of tumor cells, while TILs are a type of immune cells, reflecting the immune tumor microenvironment. Therefore, it is not surprising that RS was not highly correlated with TIL levels in our analysis.

On examining 21 genes in RS, the CD68 is the only gene obviously associated with immune function in breast cancer. CD68 is a heavily glycosylated glycoprotein that is highly expressed by macrophages and is recognized as a marker for tumor-associated macrophages [21]. In a hypothesis-generating study using human breast carcinoma treated with or without neoadjuvant chemotherapy, CD68 was found to play a role in the immune microenvironment of breast cancer [21]. Specifically, several studies using large cohorts of breast cancer patients have revealed that the expression of CD68 in tumor tissue is associated with higher grade [22, 23], increased angiogenesis [24–26], and reduced disease-free survival [25, 27, 28]. The impact of CD68 expression on the final 21-gene RS has not been of much interest to researchers. In our study, the relationship between TILs and CD68 levels remained unexplored. Hence, further studies correlating CD68 and TIL levels in ER-positive/HER2-negative cancer will shed light on the tumor-immune interaction in this subset.

There might be an association between HER2 expression and TIL levels because there is solid evidence—from translational studies using tumor samples from prospective trials—that high TIL levels correlate with response to anti-HER2 therapy [29] and that patients with HER2-positive breast cancer receiving adjuvant trastuzumab showed improved survival [30]. However, similar to real practice, we only used 21-gene RS for HER2-negative cases; hence, it might be difficult to find a link between TIL levels and HER2 expression in this study.

A growing body of evidence suggests that TIL levels are associated with the response to chemotherapy via several distinct mechanisms of the tumor-immune interaction [2–8]. Chemotherapy can increase the vulnerability of tumor cells to lytic action by cytotoxic CD8+ T cells, and selectively reduce circulating regulatory T cells as well as restore the function of natural killer (NK) cells [31]. Furthermore, the proliferation of T cells and the lytic function of NK cells were promoted by chemotherapy in breast cancer patients treated with adjuvant taxanes [32]. These findings are connected with recent studies showing that patients with lymphocyte-predominant breast cancer (LPBC) had a better prognosis compared to non-LPBC among patients treated with chemotherapy [2, 4, 6]. However, the association between TIL levels and oncologic outcome was reproducibly confirmed in TNBC, but not in ER-positive/HER2-negative breast cancer [2, 4, 6].

A meta-analysis with 1366 HR-positive/HER2-negative patients receiving NAC found that patients with high TIL levels had a worse prognosis than patients with low TIL levels, but a similar outcome compared to patients with intermediate TIL levels, even though they had a higher rate of pCR [10]. By contrast, patients with high RS had worse outcome compared to patients with low or intermediate RS [11, 12]. Taken together with our data, it appears that RS and TIL levels stratify patients into subgroups based on different tumor biology. TIL levels are relatively low in ER-positive/HER2-negative tumors than in other subtypes. Although inconsistent cut-off points (50% or 60%) have been applied to define high TIL levels, the rate of high-TIL tumors is 2.9%–13% in ER-positive/HER2-negative patients [6, 10]. Our data are in line with these findings, with a rate of high-TIL tumors of only 6.6% (13 of 198; cut-off of 60%).

The relationship between endocrine responsiveness and the degree of TILs needs to be explored. Recently, Dieci et al. evaluated the association between TIL levels in core biopsies and Ki-67 suppression after letrozole treatment with/without lapatinib for 24 weeks [33]. They found that patients with high TIL levels (cut-off of 10%) more frequently had a relative Ki-67 suppression ≥50% from baseline compared to patients with low TIL levels, without statistical significance (55% vs. 35%). Dunbier et al. showed that an inflammatory signature is associated with a poor response to 2-week aromatase inhibitor treatment, implying that TIL levels might be associated with endocrine resistance [34]. Further clinical and preclinical research should be performed.

Previously, Krishnamurti et al. compared TIL levels with RS in ER-positive cancer [35], and their study shows several differences from ours. Krishnamurti et al. did not exclude HER2-positive tumors from ER-positive tumors, whereas we only included ER-positive/HER2-negative tumors because the RS assay is applied clinically for those specific tumors. Moreover, the TIL level cut-offs are different between the studies. Krishnamurti et al. set the high TIL cut-off of as 50%, whereas we set ours at 60%, per the study by Denkert [10]. In addition, Krishnamurti et al. showed a negative correlation between TIL levels and RS, implying that TIL is a favorable marker that differs from our study. Further studies with larger cohorts are required to determine the prognostic effects of TIL levels in ER-positive/HER2-negative breast cancer.

A major limitation of our study, in addition to the short follow-up duration, is the absence of survival analyses among the groups divided by the two markers. The clinical outcomes of our study population might help refine prognostic discrimination according to these markers. Additionally, evaluation of TIL levels could differ according to the type of sample, such as core biopsies or surgical specimens. In this study, we evaluated the TIL levels using only surgically resected samples, but several studies used samples from core biopsies. Despite these limitations, our analysis comparing RS and TIL levels lays the groundwork for future research on the tumor-immune interaction in ER-positive/HER2-negative breast cancer.

Intriguingly, in our patients receiving RS-guided treatments, chemotherapy was more frequently performed among patients with high TILs and less frequently among those with low or intermediate TILs. Further studies are required considering the clinical outcomes to determine whether TIL levels could clinically benefit chemotherapy for endocrine-sensitive tumors.

## Conclusions

Despite of a very weak correlation between continuous TIL levels and RS, we found that tumors with high TIL levels tended to have a higher RS in ER-positive/HER2-negative breast cancer. Further study is warranted considering the clinical outcomes. The role of the tumor-immune interaction in ER-positive breast cancer needs to be explored in the future studies.

## Abbreviations

ANOVA: Analysis of variation
ER: Estrogen receptor
HER2: human epidermal growth factor receptor 2
HR: Hormone receptor
NAC: Neoadjuvant chemotherapy
pCR: Pathologic complete response
PR: Progesterone receptor
RS: Recurrence score
TILs: Tumor-infiltrating lymphocytes
TNBC: Triple-negative breast cancer
